# Human Pluripotent Stem Cell-Derived Cardiomyocytes for Assessment of Anticancer Drug-Induced Cardiotoxicity

**DOI:** 10.3389/fcvm.2020.00050

**Published:** 2020-04-08

**Authors:** Verena Schwach, Rolf H. Slaats, Robert Passier

**Affiliations:** ^1^Applied Stem Cell Technologies, TechMed Centre, University of Twente, Enschede, Netherlands; ^2^Department of Anatomy and Embryology, Leiden University Medical Centre, Leiden, Netherlands

**Keywords:** human pluripotent stem cells, cardiomyocytes, cardio-oncology, organ-on-chip, cardiotoxicity

## Abstract

Cardiotoxicity is a major cause of high attrition rates among newly developed drugs. Moreover, anti-cancer treatment-induced cardiotoxicity is one of the leading reasons of mortality in cancer survivors. Cardiotoxicity screening *in vitro* may improve predictivity of cardiotoxicity by novel drugs, using human pluripotent stem cell (hPSC)-derived-cardiomyocytes. Anthracyclines, including Doxorubicin, are widely used and highly effective chemotherapeutic agents for the treatment of different forms of malignancies. Unfortunately, anthracyclines cause many cardiac complications early or late after therapy. Anthracyclines exhibit their potent anti-cancer effect primarily via induction of DNA damage during the DNA replication phase in proliferative cells. In contrast, studies in animals and hPSC-cardiomyocytes have revealed that cardiotoxic effects particularly arise from (1) the generation of oxidative stress inducing mitochondrial dysfunction, (2) disruption of calcium homeostasis, and (3) changes in transcriptome and proteome, triggering apoptotic cell death. To increase the therapeutic index of chemotherapeutic Doxorubicin therapy several protective strategies have been developed or are under development, such as (1) reducing toxicity through modification of Doxorubicin (analogs), (2) targeted delivery of anthracyclines specifically to the tumor tissue or (3) cardioprotective agents that can be used in combination with Doxorubicin. Despite continuous progress in the field of cardio-oncology, cardiotoxicity is still one of the major complications of anti-cancer therapy. In this review, we focus on current hPSC-cardiomyocyte models for assessing anthracycline-induced cardiotoxicity and strategies for cardioprotection. In addition, we discuss latest developments toward personalized advanced pre-clinical models that are more closely recapitulating the human heart, which are necessary to support *in vitro* screening platforms with higher predictivity. These advanced models have the potential to reduce the time from bench-to-bedside of novel antineoplastic drugs with reduced cardiotoxicity.

## Introduction

Cardiovascular diseases and cancer are the two leading causes of death in industrialized countries. Although in recent years new anti-cancer therapies improved long-term survival rates, this is unfortunately also accompanied by an increased risk of cardiovascular complications because of adverse side effects of these anti-cancer drugs. Anthracyclines are a group of widely used anti-cancer drugs that are known to cause cardiac complications, either early or late after start of treatment (1–7). The history of anthracyclines originates in the 1950s when Daunorubicin was isolated from the bacteria *Streptomyces peucetius*. Ten years later Doxorubicin, a more effective derivative of Daunorubicin, was identified (8). However, Doxorubicin received the ominous nickname “Red Devil” because of the detrimental side effects in combination with its red color. In approximately 11% of patients, Doxorubicin-induced cardiotoxicity leads to an acute response within 2–3 days of administration (early onset of cardiotoxicity), manifested by chest pain resulting from different forms of arrhythmia (3). Chronic Doxorubicin-induced cardiomyopathy has a lower incidence (ranging from 4 to 36% dependent on the dose) and can occur as late as 10 years after the last dose (late onset of chronic cardiotoxicity), with only a 50% 1-year survival prognosis when cardiomyopathy further develops into congestive heart failure (3, 7). Importantly, anthracycline-induced cardiotoxicity endangers cancer patients since the early 70s (9, 10). At present, almost half a century later, success in preventing or counteracting anthracycline-induced cardiotoxicity has been very limited. Moreover, although novel anti-cancer therapeutic compounds to specific target molecules, such as tyrosine kinase inhibitors, have been developed, drug-induced cardiotoxicity remains a persistent problem. One major reason for this is that current *in vitro* and animal models fail to show sufficient predictive power and limit extrapolation to patients, which consequently leads to high attrition rates during the process of drug development.

Development of preclinical human cell-based models for drug discovery that mimic human physiology and pathophysiology, has the potential to improve the predictability of adverse drug events (11). In particular, human pluripotent stem cells (hPSCs) are excellent candidates for developing these models since they replicate indefinitely and have the capacity to differentiate to any cell type of the human body, including functional cardiomyocytes. This is especially embraced in the cardiac field, since isolated primary human cardiomyocytes are extremely difficult to obtain and maintain in culture. Advances in differentiation and purification of hPSC-derived cardiomyocytes (hPSC-cardiomyocytes) and their unlimited availability has promoted strategies to use these cells for cardiotoxicity assessment of new drugs (12). Previously, the Food and Drug Administration (FDA), the supreme body worldwide that checks the quality and safety of medical products, initiated the so-called “Comprehensive *in vitro* Proarrhythmia assay (CIPA),” which represents a paradigm shift and encompasses evaluation of life-threatening proarrhythmic risk for all new compounds in a preclinical assay using hPSC-cardiomyocytes (13). This CIPA initiative signifies the enormous potential of hPSC-derived cardiomyocytes for preclinical drug screening. Here, we describe underlying mechanisms of anthracycline-induced cardiotoxicity, as well as therapeutic approaches for cardioprotection. Furthermore, we will discuss the potential to use hPSC-derived cardiac models for improved safety assessment of anti-cancer drugs and strategies to overcome current limitations for developing *in vitro* drug testing platforms with a higher predictivity.

### Mechanism of Anthracycline-Induced Cardiotoxicity in hPSC-Cardiomyocytes

**Figure 1:**
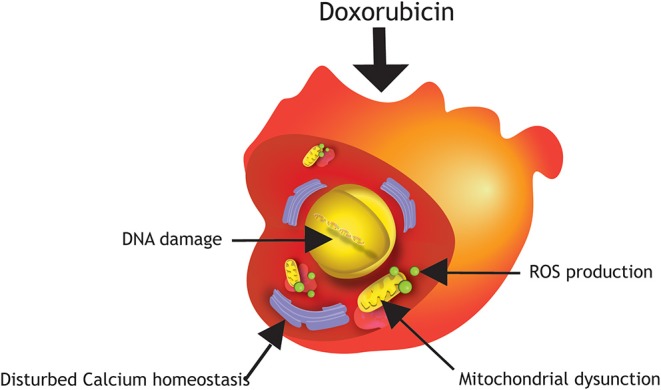
Damage mechanisms of Doxorubicin in hPSC-cardiomyocytes. Major mechanisms of anthracycline-induced cardiotoxicity in hPSC-cardiomyocytes are DNA damage, production of reactive oxygen species (ROS), mitochondrial dysfunction and disturbed calcium homeostasis.

The potent therapeutic anti-cancer effect of Doxorubicin is mediated primarily via inhibition of topoisomerase IIa, an enzyme responsible for unwinding DNA before replication or transcription. This inhibition leads to DNA damage and consequently death of highly proliferating cancer cells, but also affects healthy proliferating cells, such as hematopoietic precursors, epithelial lining of the intestine and hair follicle cells. Fully mature cardiomyocytes are typically quiescent and do not express topoisomerase IIα, however studies performed in hPSC-cardiomyocytes demonstrated that Doxorubicin may bind to the enriched topoisomerase IIβ in cardiomyocytes, and knock-out of this isotype improved cell viability significantly when exposed to Doxorubicin (14, 15). A comprehensive review on damage mechanisms of anthracycline-induced cardiotoxicity has been published elsewhere (16). The underlying mechanisms of Doxorubicin-induced cardiotoxicity is not yet completely understood. However, several studies in hPSC-cardiomyocytes have shown that mitochondrial dysfunction, disruption of calcium homeostasis, as well as altered gene and protein expression levels triggering apoptotic cell death, play important roles in anthracycline-induced cardiotoxicity (Figure 1). Increased oxidative stress by elevated production of reactive oxygen (ROS) and nitrogen species (NOS) in the mitochondria of cardiomyocytes are major factors of Doxorubicin-induced cardiotoxicity, since cardiomyocytes are more sensitive to these molecules because they possess lower levels of antioxidant enzymes than other cell types (1, 3, 4, 17–20). As a consequence, higher stress levels in cardiomyocytes under oxidative stress circumstances can lead to cardiotoxicity (20). An increase in intracellular levels of ROS and NOS cause mitochondrial dysregulation, lipid peroxidation, DNA damage, and protein carbonylation. Cardiac mitochondria are the major site of Doxorubicin-induced ROS/NOS levels due to the localization of the major redox cycling enzymes such as NAD(P)H. Additionally, Doxorubicin becomes nearly irreversibly bound to cardiolipin. Cardiolipin is an essential phospholipid, almost exclusively expressed on the inner mitochondrial membrane. It has been shown that pathological changes in cardiolipin trigger ROS production and impair mitochondrial function (21). In addition, Doxorubicin also increases mitochondrial iron accumulation which further increases ROS production in the mitochondria. Indeed, cellular and mitochondrial (as measured by cellular H_2_O_2_ production and intra-mitochondrial O^2−^ levels) ROS production in hPSC-cardiomyocytes increases already 24 h after exposure to Doxorubicin at concentrations as low as 0.01 μM (patient serum levels of Doxorubicin vary in the range of 5–10 μM after single injection). Moreover, short term exposure to 5 μM Doxorubicin induces dramatic reduction of the mitochondrial transmembrane potential indicating mitochondrial dysfunction (14, 22). To meet the high energy demand required to sustain contractile function, cardiomyocytes rely on efficient mitochondrial oxidative metabolism for energy production instead of anaerobic glycolysis. Thus, a disturbed energy metabolism is a high risk for cardiomyocyte survival. Repeated Doxorubicin exposure reduces ATP levels which cannot be restored after Doxorubicin removal suggesting a prolonged effect of Doxorubicin on energy generation in hPSC-cardiomyocytes. Together these effects pinpoint to the fact that Doxorubicin induces mitochondrial dysfunction in hPSC-cardiomyocytes (22, 23). Importantly, structural and functional disturbances are more pronounced after repeated dosing of Doxorubicin (mimicking chronic exposure) (23–25).

Another mechanism by which anthracyclines fight cancer is the induction of apoptosis. Doxorubicin effectively induces apoptotic cell death via activation of so-called death receptors (DRs), such as TNF receptor 1 (TNFR1), Fas receptor, DR4 and DR5, in many cancer types. Activation of these DRs induces the assembly of death inducing signaling complex (DISC), which starts the caspase cascade to mediate cleavage of cellular proteins and ultimately apoptosis of the cell. This DR-mediated apoptosis machinery has been shown to be conserved in human cardiomyocytes. Indeed, a Doxorubicin concentration–dependent increase of caspase 3 and 7 and Annexin V, a marker of early apoptosis, and 7-aminoactinomycin D (7–AAD) or propidium iodide as markers of late apoptosis or necrosis, suggest a strong contribution of programmed cell death to reduced cell survival after exposure to Doxorubicin. Interestingly, in hPSC-cardiomyocytes Doxorubicin induces the expression of all four DRs in a dose-dependent fashion with p53 being the key upstream activator. This suggests a p53-regulated DR-mediated apoptotic pathway a key mechanism involved in early Doxorubicin-induced cardiotoxicity. In accordance with these findings, Doxorubicin-induced apoptosis was effectively blocked by pretreating hPSC-cardiomyocytes with a DR5 neutralizing antibody. This cardiotoxic effect was reversible since expression of DR4 and 5 proteins decreased after recovery for 7 days following washout of Doxorubicin (14, 15, 22, 26, 27). In contrast to these studies with acute exposure to Doxorubicin, Li et al. showed that p53 can protect against chronic Doxorubicin exposure by counteracting mitochondrial DNA depletion after chronic exposure of hPSC-cardiomyocytes (and mice) to low doses of Doxorubicin (28).

Disturbed calcium homeostasis in cardiomyocytes hampers proper cardiac contraction. Doxorubicin has been shown to induce accumulation of intracellular calcium release from the sarcoplasmic reticulum (SR) causing calcium overload with sarcomeric disarray and myofibril deterioration in hPSC-cardiomyocytes. Moreover, Doxorubicin exposure led to downregulation of several ion channel genes, including genes that encode for calcium channels CACNs (14, 22, 26).

In summary this data shows that mitochondrial dysfunction, apoptosis, as well as disturbed calcium homeostasis are the main mechanisms of anthracycline-induced cardiotoxicity. However, in 2018, a new mechanism of Doxorubicin-induced cardiotoxicity via downregulation of the RNA-binding-protein Quaking has been identified (29). Downregulation of Quaking regulates a set of circular RNAs involved in Doxorubicin-induced apoptosis suggesting that other mechanisms cannot be excluded.

### Protective Mechanisms to Prevent or Reduce Anthracycline-Induced Cardiotoxicity

**Figure 2:**
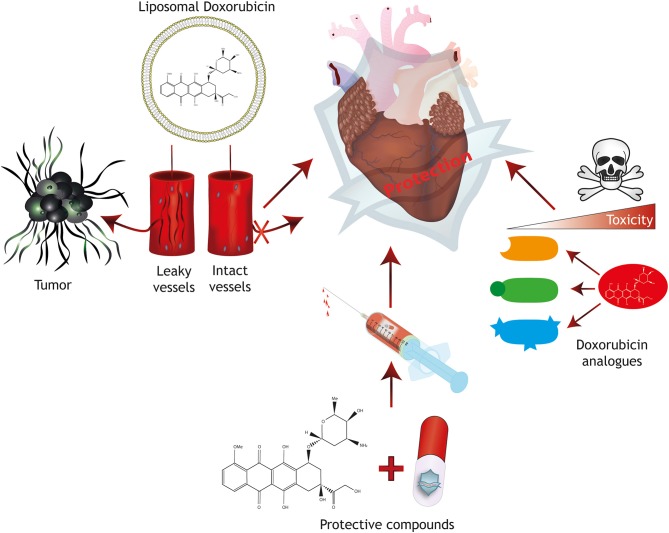
Strategies for cardioprotection. Protective mechanisms for anthracycline-induced cardiotoxicity include the generation of Doxorubicin analogs with reduced toxicity, the combined treatment with protective compounds and targeted therapy approaches including liposomal packaging of Doxorubicin. Liposomal packaging prevents Doxorubicin from exiting the vasculature in tissues with tight endothelial barriers such as the heart, but allows targeting of a tumor with leaky capillaries.

To increase the dose window of effective treatment without severe adverse side effects (therapeutic index) of conventional Doxorubicin therapy, several protective mechanisms have been developed, including modifications/analogs of Doxorubicin, targeted delivery or protective agents (Figure 2).

#### Analogs of Doxorubicin

The cardiotoxic nature of Doxorubicin pushed the development of Doxorubicin analogs into overdrive in the 1970s and 1980s, with over a 1000 analogs manufactured or discovered since (30). However, most of them failed to reach anti-tumor efficacy comparable to Doxorubicin and others were considered too toxic based on trials in rodents (30). Only a handful of analogs reached clinical trial phase, such as epirubicin, pirarubicin (also known as THP), idarubicin, mitoxanthrone, and others (31–42). The success of these analogs varies per clinical setting and malignancy, and a reduction in cardiotoxicity was not always achieved. In patients, cardiotoxic events are classically detected through loss of the left ventricular ejection fraction (LVEF), irregularities in ECG readings (in particular ST-T changes), changes in systolic time intervals (STI, indicating heart muscle failure) and in more recent work, the release of biomarkers in blood, such as cardiac Troponin-T and brain natriuretic peptide (BNP) (31, 33, 35–38, 41–45). Due to the year of discovery, early Doxorubicin analogs have not been tested *in vitro* using hPSC-cardiomyocytes to evaluate their cardiotoxicity, since these models were not available at the time. Whereas, biomarker release can also be assessed *in vitro* using hPSC-cardiomyocytes, other clinical read-outs are more difficult to evaluate *in vitro*. Nevertheless, measurement of changes in action potential, contractility, and cell survival *in vitro* may be associated with ECG/STI changes and impaired LVEF and therefore it will be of interest to test Doxorubicin analogs in hPSC-cardiomyocytes for cardiac safety.

#### Targeted Delivery Strategies

An alternative to reducing cardiotoxic properties of anthracyclines is specifically delivering anthracyclines at the location where cancer cells reside. This can be achieved by carriers that encapsulate the compound and release it at the desired location. For example, liposomal encapsulation of anthracycline inhibits transfer from the circulation to tissues with a tight endothelial barrier (e.g., the heart), but allow transfer to tissues with leaky or fenestrated vasculature, such as in tumors, but also in organs such as lung, bone marrow, lymph nodes and liver (39, 46, 47). In the last two decades, numerous trials have been conducted to assess the safety and benefit of liposome-encapsulated Doxorubicin. Success of liposomal encapsulated anthracyclines is inconsistent. Liposomal encapsulation could abolish cardiotoxicity in some clinical trials, allowing a higher cumulative dose for treatment (39, 48–50). However, in some clinical settings, liposomal Doxorubicin did not reduce cardiotoxicity compared to free anthracyclines (51), suggesting that liposomal encapsulation only has limited benefit. Unmasked liposomes such as described thus far are relatively quickly cleared from the circulation by the reticulo-endothelial system (RES), which limits the therapeutic index of so-called non-PEGylated liposomal (NPL) (39).

PEGylated liposomal Doxorubicin (PLD) features an anchored polyethylene glycol (PEG) group on the exterior lipid surface that masks them from the RES, thus reducing uptake from the circulation (52). Caelyx (Doxil), a commercialized PLD, showed significant reduction in cardiotoxicity compared to free Doxorubicin in several studies, even when used for patients who have increased risk of cardiac complications (53–55). Unfortunately, PLDs are limited in their use because of increased incidence and severity of non-cardiac side effects such as palmar-plantar erythrodysesthesia (PPE), mucositis and haematogenic disorders (54, 56). Liposomes (PEGylated or not) cannot prevent severe adverse effects of Doxorubicin entering off-target tissues. The mechanism of specific delivery of classical liposomes depends greatly on the difference in endothelial barrier permeability, which is equally high in tumors and several healthy tissues alike, and not all malignancies cohere to the “tumor with a leaky vasculature” phenotype. In an attempt to increase specific targeting of cancer cells, liposomes were conjugated with antibodies that are directed against the human epidermal growth factor receptor 2 (HER2), which is highly expressed in breast cancer cells. *In vitro*, these liposomes were effective in killing HER2-positive cancer cells, but did not affect hPSC-cardiomyocytes, highlighting their specificity (47). The HERMIONE phase II trial is now being conducted to test the safety and efficacy in patients (57). Another recent development is the encapsulation of anthracyclines in nanocages, constructed of organic (virus-like particles (VLPs), protein, DNA or carbon-based particles) or inorganic particles (supramolecular nanosystems, hybrid metal-organic, gold, or silica-based systems). Advantages of this encapsulation method are: (1) a large carrier storage capacity, (2) a targeted release of the drug at the site of interest, (3) combined with a porous structure and (4) a low immunogenic surface (58). Nanocages have not been tested in clinical trials yet, however *in vitro* data suggests improved drug targeting of cancer cells and killing efficacy, and a lower cardiotoxicity, when administered in rodents (59–63). Unfortunately, nanocages have not been specifically tested *in vitro* on hPSC-cardiomyocytes to test their cardiotoxicity.

In addition to Doxorubicin encapsulation, latest modifications to Doxorubicin are being tested on cell cultures prior to animal testing to assess their safety for the heart, using currently available hPSC-cardiomyocyte *in vitro* models. These modifications of Doxorubicin are aimed at preventing the anthracycline from exerting its toxicity on cardiomyocytes by making the drug delivery more specific. For example, DTS-201, a Doxorubicin prodrug that is injected in a stable, cell-impermeable state, is cleaved by endopeptidases that are released by tumors, enabling entry into nearby cells and specific anti-tumor activity. In a phase I trial, DTS-201 could be administered at a cumulative dose equivalent to three times the recommended dose of free Doxorubicin, without triggering a significant drop in LVEF (64). Another promising technique is the development of Doxorubicin-dendrimer conjugates with acetylgalactosamine attached to the dendrimer surface. These conjugates are specifically taken up by hepatic cancer cells, increasing therapeutic index and reducing cardiac exposure and thus toxicity in mice. In addition, *in vitro*, these conjugates did not affect hPSC-cardiomyocyte viability and electrophysiology, or induce apoptosis, demonstrating the specificity for drug uptake by the target cell type (65).

#### Protective Agents to Alleviate Cardiotoxicity

Cardioprotection during anthracycline-based therapies can also be offered by combining the anthracycline with a protective compound. As the cardiotoxic mechanism of anthracyclines involves inducing oxidative stress and mitochondrial dysfunction, protective agents that are able to lower the production of ROS or lower the workload of the heart during anthracycline treatment may be beneficial to reduce anthracycline-induced cardiotoxicity. Factors that may reduce oxidative stress in the heart, such as statins and natural antioxidants, and compounds that reduce the workload of the heart, such as angiotensin-converting-enzyme (ACE) inhibitors and beta-blockers, have been combined with anthracyclines in clinical trials, with mixed success (66–77). As far as known, these compounds have never been tested in hPSC-cardiomyocytes for assessment of their direct cardioprotective potential, even though reducing oxidative stress in particular can be readily modeled *in vitro*. Since both ischemic heart disease and anthracycline-mediated cardiotoxicity share common pathological pathways, therapies for one disease could also be beneficial for the other. Trials with more original approaches, such as remote ischemic conditioning or physical exercise and caloric restriction, strategies that were beneficial in the setting of ischemic heart disease, have been started with the purpose to reduce cardiotoxicity caused by chemotherapy (78, 79). Results of these trials are not yet available.

In hPSC-cardiomyocytes it has been shown that Doxorubicin causes arrhythmic contractions by inducing the accumulation of calcium in the cell and thereby disturbing calcium homeostasis (14, 22). This would suggest that calcium antagonists can help to reduce Doxorubicin-induced cardiotoxicity. Interestingly, a calcium antagonistic compound called prenylamine, was able to reduce cardiotoxic effects when co-administered with Doxorubicin, before it was withdrawn from the market 1 year later (80). Reason for withdrawal was the pro-arrhythmic properties of prenylamine, inducing long QT syndrome (81). Other calcium agonists are currently primarily used at lower doses to reduce Doxorubicin resistance of cancer cells, by inhibiting their capacity to pump out drugs via the P-glycoprotein ATP-dependent efflux pumps (82). Low doses of these calcium antagonists could be repurposed to reduce Doxorubicin-induced cardiotoxicity.

Neuregulin-1β, an ErbB receptor (HER2) family ligand, has been proven effective against Doxorubicin-induced cardiotoxicity, but is also pro-neoplastic in many cancers via formation of ErbB2/3 interactions (83–86). Even if neuregulin, as key mediator of endothelial–cardiomyocyte crosstalk, is able to protect ventricular cardiomyocytes from anthracycline-induced apoptosis, its use might thus be controversial in the clinic. Nevertheless, phase I, II and III clinical trials have been completed or are ongoing (NCT01251406, NCT1214096, and NCT01541202) (84–86). Patients with symptomatic heart failure (HF) and left ventricular dysfunction showed improved cardiac function with increased LVEF after treatment with recombinant human neuregulin 1. In contrast to native neuregulin-1β, engineered bivalent neuregulin-1β has reduced pro-neoplastic potential because of a shift toward ErbB3 homotypic interactions. Bivalent neuregulin-1β is anti-neoplastic or cytostatic in cancer cells. Importantly, bivalent neuregulin-1β showed similar cardioprotective properties as neuregulin in hPSC-cardiomyocytes and in mice with chronic cardiomyopathy (87). Especially the reduced pro-neoplastic potential of bivalent neuregulin-1β offers translational potential for cardioprotection after anthracycline therapy, however to date, bivalent neuregulin-1β has not been taken into clinical trial.

Dexrazoxane, a catalytic topoisomerase inhibitor and iron chelator, is the only marketed cardioprotective agent that is used specifically to counteract the adverse effects of anthracyclines (88). In children with acute lymphoblastic leukemia, leukemia or lymphoma, the combined treatment with Doxorubicin and Dexrazoxane significantly reduces release of troponin (as biomarker of cardiotoxicity) and Doxorubicin-induced cardiac remodeling. Importantly, the combination with Dexrazoxane improves left ventricular function compared to Doxorubicin alone (89, 90). Dexrazoxane is also effective in relapsed patients with leukemia when administered prior or after a high cumulative dose (91). In breast cancer patients with increased risk of cardiotoxicity because of an enhanced cumulative dose of anthracycline, Dexrazoxane also robustly reduced the incidence of cardiac events, such as decreased ejection fraction, and incidence and severity of CHF (92, 93). Even in a retrospective 2–20 year follow-up, Dexrazoxane demonstrated late-clinical and subclinical cardioprotective effect (94).

Treatment of cardiomyocytes with Dexrazoxane is therefore expected to prevent cardiotoxicity. However, pre– and co–treatment of hPSC-cardiomyocytes with Dexrazoxane could not alleviate Doxorubicin-induced cardiotoxicity and increased cardiotoxicity at concentrations as low as 0.1 μM (22, 95). This discrepancy between *in vivo* and *in vitro* may be related to the relative immature character of hPSC-cardiomyocytes. Early stage hPSC-cardiomyocytes may be more sensitive to Doxorubicin because of higher expression levels of topoisomerase IIα and are thus more prone to severe DNA damage (as mentioned earlier: Doxorubicin primarily affects topoisomerase IIα), whereas in mature cardiomyocytes topoisomerase IIα switches to the IIβ isoform. Importantly, Dexrazoxane might exert its protective effects via depletion of topoisomerase IIβ, the major topoisomerase isoform in more mature cardiomyocytes (95, 96). This suggests that more advanced models are needed to assess cardiotoxicity *in vitro*.

### HPSC-Cardiomyocyte Models in Drug Screening of Tyrosine Kinase Inhibitors (TKis)

Not only anthracyclines, but also safety assessment of other classes of anti-cancer drugs may benefit from hPSC-based *in vitro* models. For example, malignancies caused by hyperactive receptor tyrosine kinases (RTKs), which drive proliferation and survival, are treated with FDA-approved tyrosine-kinase inhibitors (TKis). However, a number of TKis have been associated with adverse cardiac side effects following treatment of cancer patients, such as reduced LVEF, myocardial infarction, arrhythmias and heart failure (97). While animal models have often failed to predict cardiotoxic effects during safety assessment (98), TKis have shown to inhibit viability, contractility, electrophysiology, calcium handling and cardiac oxidative phosphorylation of hPSC-cardiomyocytes (99–101). Different TKis show cell type specific cytotoxicity, either in hPSC-derived cardiomyocytes, endothelial cells and fibroblasts or hPSCs, suggesting that TKis differently affect cardiovascular and non-cardiovascular cell types (99).

TKis can be subdivided into small molecules, including crizotinib, sunitinib, or sorafenib, and monoclonal antibodies. Importantly, different TKis have distinct toxicity profiles and not all TKIs induce cardiotoxicity (97). The broad-range TKi sunitinib, for example, decreased hPSC-cardiomyocyte viability, as well as AMP-activated protein kinase (AMPK), increased lipid accumulation and induced arrhythmic events in sunitinib-treated hPSC-cardiomyocytes. Crizotinib, an ALK/MET inhibitor, increased ROS production and caspase activation, induced cholesterol accumulation and an irregular beat pattern. Similarly, Nilotinib, a second generation Bcr-Abl inhibitor, increased ROS generation and caspase activation and induced arrhythmic beating. Interestingly, compared to the TKis sunitinib, crizotinib and nilotinib, the relatively cardiac-safe TKi erlotinib only displayed minor effects on hPSC-cardiomyocyte health in the same study (98) which suggests that hPSC-cardiomyocytes may represent a reliable *in vitro* model for predicting cardiotoxic side effects.

RTK activity may also be reduced by blocking the human epidermal growth factor receptor 2 (HER2), which activation is a common feature of a subset of malignant diseases, particularly breast cancer (102). While trastuzumab (TZM), a TKi monoclonal antibody blocking HER2, greatly improves treatment against HER2 positive malignancies, inhibition of HER2 signaling was found to be detrimental for cardiac function (102–104). In a subset of breast cancer patients, treatment with TZM led to mild to severe decrease in LVEF without apparent myocardial tissue damage or cell loss. Using patient-derived hPSC-cardiomyocyte 2D monolayer cultures it was shown that TZM affected metabolic and mitochondrial processes, of which the severity depended on genetic variation. Moreover, this disease phenotype could be rescued using metabolism-stimulating agents, such as AMPK (102). Additionally, HER2 activation triggered by exogenous neuregulin-1 could reduce hPSC-cardiomyocyte damage and cell loss during Doxorubicin exposure, an effect that was lost upon inhibition of the HER2 receptor by TZM (103, 104). These findings indicate that hPSC-cardiomyocyte 2D models may serve as a valuable tool to identify cardioprotective compounds in a high throughput system, which can be further validated in more advanced human-based 3D cardiac models.

### Generation of Advanced hPSC-Derived *in vitro* Models for Accurate Assessment of Cardiac Safety

**Figure 3:**
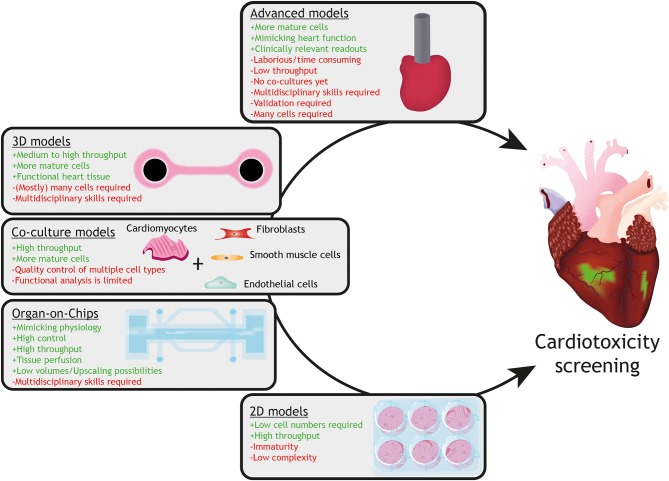
Current *in vitro* models with hPSC-cardiomyocytes and their advantages (+ green) and limitations (– red). Models with increasing complexity (from 2D monolayers to 3D advanced models) are available for assessment of drug-induced cardiotoxicity utilizing hPSC-cardiomyocytes. Whereas 2D monolayer cardiomyocyte cultures can be used for high throughput-screening for specific cellular responses, they lack the physiology, multicellular interactions and tissue organization, which can be provided by organ-on-chip, co-culture or 3D tissue models (for instance engineered heart tissues). Advanced cardiac models, such as an engineered mini heart tube with the capacity to pump fluid, mimic clinically-relevant function(s) of the heart.

Although cardiotoxic effects could be observed in hPSC-cardiomyocyte based models, not all aspects of *in vivo* cardiotoxicity are recapitulated in current human *in vitro* models. One of the important shortcomings of hPSC-cardiomyocyte models is their relative immature phenotype. Reviews on the topic of the immaturity of hPSC-cardiomyocytes, including strategies to increase the level of maturity have already been described elsewhere (105–107). Figure 3 shows a description of current *in vitro* models utilizing hPSC-cardiomyocytes, including their advantages and limitations. It is therefore crucial to develop innovative advanced human models that approximate the adult human heart. In order to achieve this, defined multicellular models, consisting of the main cell-types of the heart, such as atrial or ventricular cardiomyocytes, endothelial cells, smooth muscle cells and fibroblasts, need to be constructed.

Cardiac tissue formation in 3D tissues, such as cardiac microtissues, more closely mimics native heart tissue which might allow studying more physiologically relevant dosing profiles and deciphering acute vs. chronic cardiotoxicity (25). For generating other 3D advanced cardiac models, so-called engineered heart tissues (EHTs), hPSC-cardiomyocytes are mixed with fibroblasts (or other cardiac cells) in a hydrogel and poured into a casting mold around silicon posts, leading to beating structures within the first week under continuous mechanical strain. Previously, it has been shown that 3D EHTs display a higher degree of maturation based on sarcomeric organization, formation of T-tubules and functional characterization (e.g., contraction force, electrophysiology) (108–110). Cardiotoxicity of anti-cancer agents, other than anthracyclines, such as the TKi sunitinib, has already been assessed in EHTs, which resulted in triggered activation of apoptosis and loss of contractile force, spontaneous beating and mitochondrial membrane potential (111).

Until now studies did not consider subtype-specific cardiomyocytes for cardiotoxicity testing. This would be of high importance, as different cardiac subtypes may exhibit different susceptibility to Doxorubicin. Especially, because of the well-established arrhythmogenic potential of Doxorubicin (31, 112, 113), it is highly relevant to assess arrhythmogenic effects of Doxorubicin on hPSC-derived atrial and ventricular cardiomyocytes. Moreover, in a trial comparing Doxorubicin to Epirubicin in treatment of breast cancer, a quarter of all cardiac side effects of Doxorubicin was identified as sinus tachycardia, for Epirubicin this was half of the cardiotoxic responses in the trial population (31). In addition, a recent study showed that genetic loci are associated with both increased sensitivity to Doxorubicin-induced hPSC-pacemaker cell death and a higher risk of arrhythmia in patients (114), which justifies to perform cardiotoxicity testing on pacemaker cells. Previously, we and others have shown efficient production of atrial, ventricular and pacemaker cardiomyocytes from hPSCs (115–118).

In the cardiac microenvironment *in vivo*, each cardiomyocyte is surrounded by 3–4 capillaries with a distance between each cardiomyocyte and an endothelial cell of about 1 μm (119). This delicate build-up allows for defined cardiomyocyte-endothelium crosstalk via paracrine signaling and cell-cell contact (119–122). This data suggests that the crosstalk of human cardiomyocytes with highly metabolic active cardiac endothelial cells is very important. Recent advances in micro-engineering and stem cell technologies enabled development of microfluidic devices in which living cells (mostly several cell-types) are cultured in channels or small compartments and perfused in a controlled manner, mimicking the microenvironment and responses of tissues or organs (123–125). These so-called *organ-on-chips* can be combined with integrated sensors (for example for biochemical and electrical readouts) and are compatible with live imaging (for example using stem cell-based fluorescent reporter lines) (126) and human cell and tissue sampling. Very recently, Weng et al. (127) generated a multiple chambered tissue chip, a so-called organ-on-a-chip, which allowed simultaneous culturing of tumor tissue and hPSC-cardiomyocytes while being connected via a microfluidic channel, lined with endothelial cells. This design enables simultaneous testing of potential anti-tumor effects and cardiotoxicity of drugs administered via the endothelial layer. Another important aspect of drug efficacy and safety assessment is to predict pharmacokinetic parameters, determined by absorption, distribution, metabolism and excretion (ADME) of drugs, which are difficult to measure or model *in vitro*. As mentioned before, organ-on-chips mimic organ-like function and responses in perfusable mircophysiological systems and may offer an opportunity to overcome current limitations related to pharmacokinetic modeling. Multiple organ-on-chip devices with organ-level functionality could be connected to each other with vascularized, endothelium-lined channels mimicking blood circulation and recapitulating tissue-tissue interfaces, thus modeling organ crosstalk and metabolism of compounds in a single system (128, 129). For predictive physiologically based pharmacokinetic (PBPK) modeling, it is of paramount importance to combine defined hPSC culture methods and 3D tissue engineering or organoid formation with technical advances in the field of organ-on-chip technology, ultimately leading to interconnected multiple human organ-on-chip platforms (for example, combining heart-, liver- and gut-on-chip models).

## Future Outlook

Because of the previously mentioned shortcomings, a full recapitulation of clinical cardiotoxic manifestations is beyond the capacity of current *in vitro* models. Interestingly, recent developments have shown that it is feasible to build human *in vitro* models that are more closely resembling functional human hearts for assessment of clinically relevant parameters, which may greatly enhance the predictability of these *in vitro* models for safety pharmacology and disease phenotypes. Recently, Li et al. created for the first time a human ventricular-like cardiac organoid chamber (hvCOC) with hPSC-cardiomyocytes and dermal fibroblasts (130). Similarly, Macqueen and colleagues generated a one cell layer thick ventricular-like tube from hPSC-cardiomyocytes (131). Both HvCOCs and ventricular tubes were able to pump fluid, which allowed measuring ejection fraction and pressure-volume loops as readouts for cardiac function. Although these developments are promising, combination with other cardiac cells, such as endothelial and smooth muscle cells and fibroblasts, may lead to further maturation and improvement of function. Future studies will be required in order to evaluate the predictive values of these human advanced 3D cardiac models in preclinical drug testing, their potential to reduce time for bringing new drugs from bench-to-bedside and repurposing of drugs.

Moreover, state-of-the-art multiple organ-on-chip platforms will also advance predictability of efficacy and toxicity of combinatorial drug (cardioprotective) treatment and specific target delivery.

It is evident that interpatient variability further complicates development of predictive *in vitro* models for drug screening. This is also true for anthracycline-induced cardiotoxicity, which varies tremendously from patient to patient. These variations may be caused due to environmental factors, life-style or genetic variance. Patient-derived (or genetically modified) hPSCs provide the possibility to include disease-associated genetic risk factors in these *in vitro* models, which can be directly compared to isogenic control hPSC lines. Interestingly, studies with hPSC-cardiomyocytes have shown that cardiotoxic effects of Doxorubicin were dependent on the genetic background of patient-derived hPSC-cardiomyocytes (22). Similarly, characterization of hPSC-cardiomyocytes from 45 individuals showed inter-individual variation in transcriptional response after 24 h exposure to different concentrations of Doxorubicin, which were predictive of *in vitro* cell damage as measured by cardiac troponin release of cardiomyocytes (132). These findings highlight the importance of personalized medicine and risk stratification, which will allow us to predict for which patients new therapies will be effective and safe and for which not. This will reduce the attrition rate, costs and time of drug development and facilitate repurposing of drugs that have shown a lack of efficacy or risk of toxicity. Consequently, successful implementation of these advanced hPSC-based models will lead to better and safer drugs.

## Author Contributions

All authors listed have made a substantial, direct and intellectual contribution to the work, and approved it for publication.

### Conflict of Interest

The authors declare that the research was conducted in the absence of any commercial or financial relationships that could be construed as a potential conflict of interest.
